# High-flow nasal cannula therapy in pediatric care: A regional practice survey from KSA

**DOI:** 10.1016/j.jtumed.2026.04.007

**Published:** 2026-05-12

**Authors:** Ali M. Alasmari

**Affiliations:** Respiratory Therapy Department, College of Medical Rehabilitation Sciences, Taibah University, Madinah, KSA

**Keywords:** Clinical practice, High-flow nasal cannula, Madinah, Pediatric care, Respiratory therapy, Western KSA, القنية الأنفية عالية التدفق, رعاية الأطفال, العلاج التنفسي, الممارسة السريرية, المدينة المنورة, غرب المملكة العربية السعودية

## Abstract

**Objectives:**

To describe current practices related to high-flow nasal cannula (HFNC) therapy among respiratory therapists (RTs) working in pediatric settings across the western region of KSA, specifically in the cities of Almadinah Almunawwarah, Jeddah, and Makkah.

**Methods:**

Between January and April 2025, a cross-sectional survey was distributed online to RTs working in pediatric inpatient units in the three cities. The validated questionnaire collected data regarding demographics, hospital characteristics, HFNC initiation practices, adjustment methods, assessment routines, and aerosol delivery practices.

**Results:**

In total, 102 RTs from 10 government hospitals participated in the study, where 51.9% worked in standalone children's hospitals. HFNC was most commonly initiated for pneumonia (79.4%) and bronchiolitis (73.5%). Definitions of HFNC varied where 65% of respondents described it as heated gas delivered at a flow rate equal to or exceeding the patient's inspiratory demand. Initial settings were determined based on patient weight (44.1%), therapist-driven protocols (38.2%), or age (9.8%). When oxygenation was suboptimal, 73.5% of RTs increased the FiO_2_ and 26.5% increased the flow. In cases of HFNC failure, 89.2% transitioned patients to noninvasive ventilation (bilevel positive airway pressure/continuous positive airway pressure) and 10.8% opted for intubation. Aerosol therapy was administered via HFNC by 88.2% of respondents and 50% used vibrating mesh nebulizers. During aerosol delivery, 4.90% of RTs reduced HFNC flow and 29.4% temporarily discontinued HFNC.

**Conclusion:**

HFNC is commonly used in pediatric care across the western region of KSA. However, our results emphasize the need to establish national guidelines or protocols for pediatric HFNC therapy to ensure standardized care and efficient resource utilization.

## الملخص

**أهداف البحث:**

وصف الممارسات الحالية لعلاج القنية الأنفية ذات التدفق العالي (HFNC) بين أخصائيين العلاج التنفسي (RTs) في أقسام الأطفال بالمنطقة الغربية من المملكة العربية السعودية، وتحديداً في مدن المدينة المنورة وجدة ومكة المكرمة.

**طرق البحث:**

تم توزيع استبانة مقطعيه عبر رابط الإنترنت خلال الفترة من يناير إلى أبريل 2025 على أخصائيين العلاج التنفسي العاملين في وحدات المرضى الداخليين للأطفال في المدن الثلاث مدن المدينة المنورة وجدة ومكة المكرمة. الاستبانة جمعت بيانات حول الخصائص الديموغرافية، وخصائص المستشفى، وممارسات بدء استخدام HFNC، وطرق التعديل، وروتين التقييم فاعلية الجهاز على المرضى، وممارسات توصيل العلاج بالرذاذ عبر HFNC.

**النتائج:**

شارك ما مجموعه 102 أخصائي علاج تنفسي من 10 مستشفيات حكومية؛ حيث يعمل 51.9% منهم في مستشفيات الأطفال والولادة. كان ال HFNC يُستخدم بشكل شائع بين المرضى الأطفال المنومين لعلاج امراض الالتهاب الرئوي (79.4%) والتهاب القصيبات الهوائية (73.5%). اختلفت تعريفات HFNC، حيث وصفه 65% بأنه غاز مُسخَّن يُقدَّم بمعدل تدفق يساوي أو يزيد عن طلب الشهيق للمريض. افاد المشاركين في الاستبانة بأنه يتم تحديد الإعدادات الأولية بناءً على وزن المريض (44.1%)، أو بروتوكولات متبعه من قسم العلاج التنفسي (38.2%)، أو عن طريق حساب عمر المريض (9.8%). و افاد معظم المشاركين انهم عند نقص الاكسجين في الدم يقوم 73.5% بزيادة تركيز الأكسجين أولا (FiO^2^) في جهاز HFNC ، بينما يفضل الاخرين 26.5% زيادة معدل التدفق. وعند سؤالهم في حال فشل العلاج بـ HFNC، فضل 88.2% الانتقال إلى التهوية غير الغازية، في حين اختار 10.8% التنبيب. في حين تم توصيل العلاج بالرذاذ عبر HFNC من قبل 88.2%، حيث استخدم 50% منهم أجهزة رذاذ شبكية اهتزازية. وخلال توصيل الرذاذ، خفّض 13% معدل تدفق HFNC مؤقتاً، وأوقفه 23% بشكل مؤقت.

**الاستنتاجات:**

أظهر أخصائيين العلاج التنفسي استخداما عاليا HFNC، في حين تظهر الدراسة تباينات كبيرة في ممارسات HFNC بين أخصائيين العلاج التنفسي في المنطقة الغربية من المملكة العربية السعودية. لذلك، يُعد استحاث إرشادات موحدة قائمة على الأدلة العلمية أمراً ضرورياً لدعم استخدام HFNC من قبل الأخصائيين، وتعزيز سلامة المرضى، وضمان نتائج سريرية أكثر اتساقاً.

## Introduction

High-flow nasal cannula (HFNC) therapy is a respiratory support system that delivers humidified and heated oxygen through nasal prongs at high flow rates, ranging from 2 to 60 L/min.^1^ In recent years, the use of HFNC therapy has expanded as an alternative to noninvasive ventilation for managing respiratory distress in pediatric care, largely due to ease of use and favorable patient tolerance.^2^ Compared with other noninvasive ventilation modalities, HFNC has several physiological advantages, including heated humidification, anatomical dead space reduction, improvement in the work of breathing, and heart rate and respiratory rate decreases.^3^^,^^4^

Clinically, HFNC management was initially limited to pediatric intensive care units (PICUs); however, its use has since expanded to emergency departments, inpatient pediatric wards, and even home care settings.^5^ Studies have demonstrated that delivering humidified gas via HFNC reduces upper airway resistance in the nasal mucosa caused by dry, cold gas and enhances mucociliary clearance.^6^^,^^7^ In pediatric populations, HFNC has been shown to generate positive end-expiratory pressure, promote anatomical dead space washout, and reduce the work of breathing by minimizing CO_2_ rebreathing.^8^^,^^9^ Acute bronchiolitis is one of the most common pediatric respiratory conditions and meta-analyses have shown that treatments with both HFNC and continuous positive airway pressure (CPAP) are superior to conventional oxygen therapy.^10^ A retrospective study in KSA further found that 75% of children admitted to a PICU with acute respiratory distress were treated with HFNC, resulting in significant improvements in the respiratory rate, heart rate, systolic blood pressure, and oxygen saturation.^11^ However, randomized controlled trials involving pediatric patients remain limited,^12^ and a recent Cochrane review was unable to provide evidence-based recommendations due to a lack of qualifying studies.^13^ Thus, despite its increasing use, there is still a clear lack of evidence or consensus-based recommendations regarding when to initiate or how to wean HFNC therapy.^14^

Respiratory therapists (RTs) play a critical role in optimizing oxygen delivery for pediatric patients by administering oxygen therapy through various modalities, including HFNC.^15^ At present, national guidelines have not been published regarding the use of HFNC therapy in the management of pediatric conditions in KSA. Several cross-sectional studies have reported increasing reliance on HFNC; however, these studies also highlight a lack of well-established policies or guidelines to govern its use among RTs, particularly in neonatal critical care settings within the country.^16^, ^17^, ^18^, ^19^ Therefore, it is clearly necessary to assess how RTs implement HFNC therapy in pediatric care settings across the western region of KSA. It was hypothesized that HFNC practices would vary significantly among RTs working in pediatric units in this region. Accordingly, in this study, the aim was to describe current HFNC use by RTs in pediatric units across the major western cities of KSA, specifically Almadinah Almunawwarah, Jeddah, and Makkah.

## Materials and Methods

### Study design

This observational cross-sectional study was based on an online questionnaire distributed to RTs in KSA between January and April 2025. The study protocol received exempt approval from the College of Medical Rehabilitation Sciences Research Ethics Committee at Taibah University (CMRS-REC2025-0016). All participants provided electronic informed consent prior to participation. The reporting of data in this study adhered to the Strengthening the Reporting of Observational Studies in Epidemiology (STROBE) checklist.^20^

### Participants, sampling, and recruitment process

This survey targeted all governmental hospitals with inpatient pediatric units located in the major cities in the western region of KSA, i.e., Almadinah Almunawwarah, Jeddah, and Makkah. The study population consisted of RTs holding a valid license in KSA and actively practicing in pediatric units. Undergraduate RT students in their internship year were excluded because the study aimed to investigate clinical decision making by trained RTs. Among the 14 governmental hospitals in the region with pediatric units, RT staff from 10 hospitals completed the survey. Two follow-up attempts were made with department managers after the initial distribution of the survey. Units that did not respond after both follow-up attempts were classified as non-respondents in the analysis.

Convenience sampling combined with a snowballing technique was employed.^21^ To facilitate recruitment, a survey link was initially distributed to RT department managers at the selected hospitals, who were asked to share the survey through internal professional networks and departmental communication channels. This approach was selected to maximize reach and response rates while ensuring participation from RTs with direct clinical experience in pediatric patient management. Potential participants received an invitation in English describing the study's purpose and containing a link to the questionnaire hosted on Google® Forms.

### Addressing potential sources of bias

Due to the recruitment strategy, RTs with greater interest in HFNC therapy or higher confidence in their knowledge may have been overrepresented. In addition, the use of a snowball sampling method, which is a form of convenience sampling, may have introduced selection bias and limited the representativeness and generalizability of the findings. Consequently, the study outcomes should be interpreted with appropriate caution.

### Data collection instrument

This study employed a previously validated survey instrument designed to assess HFNC practices by RTs for pediatric patients in the United States.^19^ Permission was obtained to use the survey tool. The original instrument was developed following comprehensive literature review and the content validity was evaluated by a panel of five RT experts with experience in HFNC use and survey research. The panel reviewed the instrument and recommended modifications related to wording, format, and content. Following these revisions, the panel determined that the survey was suitable for assessing current HFNC practices among RTs caring for pediatric patients.

The survey collected data regarding sociodemographic and hospital characteristics, including age, gender, academic level, years of work experience, city, and hospital type. A second section assessed the initial HFNC flow settings for RTs, adjustment practices, locations where HFNC was used, frequency of assessments, parameters evaluated during assessments, and methods used for aerosol delivery via HFNC when applicable.

### Data analysis

Descriptive analyses were conducted, where continuous variables were presented as means and standard deviations or medians and interquartile ranges, as appropriate, whereas categorical variables were summarized using frequencies and percentages. Data analysis was performed using SPSS® version 27.0 (IBM, Chicago, IL, USA).

## Results

### Participant and unit characteristics

In total, 132 survey responses were collected. Nine were excluded due to excessive numbers of missing or incomplete responses, and 21 more were excluded as they did not meet the inclusion criteria (undergraduate internship students). The final analytic sample comprised 102 responses. Among the 102 respondents, approximately two-thirds were male. A slightly higher proportion of participants worked in standalone children's hospitals (51.9%) compared with tertiary or academic centers (48.1%). Ten governmental hospitals with pediatric units in the western region participated in the study, with half of these hospitals located in Jeddah. Most participating hospitals (60.0%) reported that their pediatric units had implemented clinical policies or guidelines, as shown in Table 1.

**Table 1:** Participant demographics and hospital characteristics.

Demographic Variable	n (%)
Total respondents (n)	102
Respondents from standalone children's hospitals	53 (51.9)
Respondents from tertiary/academic hospitals	49 (48.1)
Age, median [IQR]∗	25 [22–52]
Gender	
Male	68 (66.7)
Female	34 (33.3)
Educational level	
Associate degree	1 (1.0)
Bachelor's degree	90 (88.2)
Master's degree	11 (10.8)
Work experience	
< 1 year	39 (38.2)
1–4 years	40 (39.2)
5–10 years	13 (12.7)
> 10 years	10 (9.8)
Number of participating hospitals:	10
Participating hospitals by city	
Almadinah Almunawwarah	3 (30.0)
Jeddah	5 (50.0)
Makkah	2 (20.0)
Hospitals with implemented HFNC∗ clinical policies/guidelines	6 (60.0)
Reported hospital locations for HFNC use	
Emergency department	21 (20.6)
General pediatric wards	13 (12.7)
PICU∗	68 (66.7)

∗Abbreviations: HFNC, high-flow nasal cannula; IQR, interquartile range; PICU, pediatric intensive care unit.

### Patient characteristics and parameters monitored during HFNC

Most RT respondents reported that HFNC was most frequently initiated for pediatric patients with pneumonia (79.4%), bronchiolitis (73.5%), and asthma (62.7%). The most commonly monitored parameters during HFNC therapy were pulse oximetry (94.1%), respiratory rate (92.2%), and signs of increased work of breathing (91.2%), whereas near-infrared spectroscopy was rarely used (2.0%), as shown in Table 2.

**Table 2:** Patient characteristics and parameters monitored during HFNC use.

Patient Characteristics	Variable Respondents, n (%)
Conditions for which HFNC is used	
Bronchiolitis	75 (73.5)
Asthma	64 (62.7)
Pneumonia	81 (79.4)
Postoperative respiratory support	30 (29.4)
ARDS	44 (43.1)
Parameters monitored during RT assessments	
Respiratory rate	94 (92.2)
Heart rate via continuous ECG	44 (43.1)
Work of breathing	93 (91.2)
Blood pressure	19 (18.6)
Pulse oximetry (SpO_2_)	96 (94.1)
Transcutaneous CO_2_	19 (18.6)
Near infrared spectroscopy	2 (2.0)

∗Abbreviations: ARDS, acute respiratory distress syndrome; ECG, electrocardiogram; HFNC, high-flow nasal cannula; RT, respiratory therapist.

**Figure 1:**
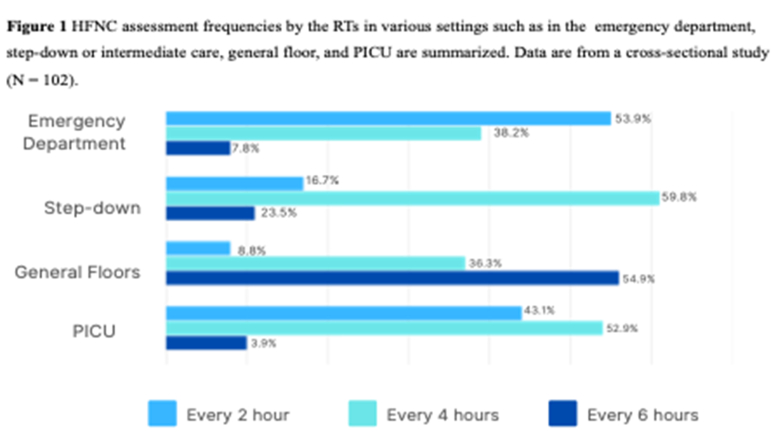

Figure 1 summarizes the frequency of clinical assessments performed by RTs during HFNC initiation across various hospital settings, including emergency departments, step-down or intermediate care units, general pediatric wards, and PICUs.

### Perspectives of participants on definition, setup, and adjustment of HFNC

As shown in Table 3, nearly two-thirds of RTs defined HFNC as heated gas delivered through a nasal cannula at flow rates that meet or exceed the patient's inspiratory demand. At therapy initiation, 44.1% of RTs reported using a weight-based approach and 38.2% followed RT-driven protocols. Changes in vital signs were cited as the most common reason for adjusting flow by 64.7% of respondents. In cases of hypoxia, increasing FiO_2_ was the most frequently reported intervention (73.5%). When HFNC failed to improve the patient's condition, the majority of RTs (89.2%) transitioned patients to noninvasive ventilation using bilevel positive airway pressure (BiPAP) or CPAP.

**Table 3:** Perspectives of participants on definition, setup, and adjustment of HFNC.

Survey Item	Respondents, n (%)
A. How does your facility define HFNC?	
Any heated, humidified, and blended gas delivered via nasal cannula	16 (15.7)
Heated gas delivered via nasal cannula at flow ≥ patient inspiratory demand	66 (64.7)
Nasal cannula flow above predefined thresholds based on age or weight	20 (19.6)
B. Techniques used to establish the initial flow rate	
Formula based	8 (7.8)
RT-driven protocol	39 (38.2)
Weight based (e.g., 1 L/kg/min)	45 (44.1)
Age based	10 (9.8)
C. Flow adjustment	
RT-driven protocol	23 (22.5)
Based on vital signs	66 (64.7)
Per physician orders	13 (12.7)
D. If oxygenation on HFNC is below target, what is the first intervention?	
Increase FiO_2_	75 (73.5)
Increase flow	27 (26.5)
E. Next step if the patient fails HFNC	
Noninvasive ventilation	68 (66.7)
CPAP	23 (22.5)
Intubation	11 (10.8)

∗Abbreviations: CPAP, continuous positive airway pressure; FiO_2_, fraction of inspired oxygen; HFNC, high-flow nasal cannula; RT, respiratory therapist.

### Administration of aerosolized medications during HFNC

Practices related to aerosolized medication delivery during HFNC therapy are summarized in Table 4. Approximately half of the RTs reported administering aerosol treatments within the HFNC circuit using a vibrating mesh nebulizer. The most commonly delivered medications were β_2_-adrenergic receptor agonists. Nearly half of respondents (48%) indicated that the nebulizer was positioned on the dry side of the humidification circuit during administration.

**Table 4:** Aerosol therapy practices and medications administered during HFNC.

Survey Item	Respondents, n (%)
A. Delivering aerosol therapy to patients receiving HFNC∗ (yes/no)	90 (88.2)
B. When delivering aerosolized medications to patients receiving HFNC do you (select all that apply):	
Deliver aerosol through HFNC setup with vibrating mesh nebulizer	51 (50.0)
Deliver aerosol through HFNC setup with jet nebulizer	41 (40.2)
Take patient off HFNC and deliver treatment with MDI or jet nebulizer	30 (29.4)
Reduce HFNC flow	5 (4.90)
C. What medications do you deliver via HFNC (select all that apply):	
β_2_-adrenergic receptor agonists (e.g., albuterol, levalbuterol, salbutamol)	83 (92.2)
Mucolytics (dornase alfa, acetylcysteine, hypertonic saline)	37 (41.1)
Corticosteroids (e.g., budesonide)	51 (56.9)
D. Where do you place the nebulizer in the HFNC setup:	
On the dry side of the humidifier	43 (48.0)
On the wet side of the humidifier	9 (10.8)
Between the circuit and nasal cannula and circuit	37 (41.2)

∗Abbreviations: HFNC, high-flow nasal cannula; MDI, metered-dose inhaler.

## Discussion

This regional study is the first to show that HFNC therapy is widely utilized in pediatric care units in KSA but practices remain heterogeneous. The present study was descriptive and cross-sectional, so our findings should be interpreted as evidence of variation in practice rather than as evidence that any specific HFNC strategy improves or worsens clinical outcomes. Thus, our findings align with recent national and international surveys that demonstrated persistent between-center and between-clinician variability in how HFNC is defined, initiated, titrated, and reassessed, even in settings where local or national guidance exists.^16^, ^17^, ^18^, ^19^

A crucial observation in our data set was the lack of uniformity in terms of both the definition of HFNC and approach used to determine initial flow and subsequent adjustments. Clinically, the physiological effects of HFNC therapy depend on the delivered flow, inspiratory demand of the patient, cannula fit and leak, humidification, and interface setup.^5^ Accordingly, this observation is clinically relevant because variations in how clinicians define “high flow” may reasonably affect the magnitude of dead-space washout, reductions in work of breathing, and patient comfort.^22^ Therefore, our findings probably reflect differences in the preferences of individual clinicians and how institutions conceptualize HFNC within local workflows and protocols.^23^

Existing pediatric evidence suggests a more cautious interpretation of the effectiveness of HFNC,^2^ with the strongest data derived from infants with bronchiolitis. A Cochrane review conducted in 2024 indicated that compared with low-flow oxygen, HFNC may decrease the need to escalate treatment, slightly lower respiratory and heart rates, and shorten the length of hospital stay and duration of oxygen therapy.^24^ However, the confidence in several of these outcomes was only low to moderate, and adequate evidence remains lacking to compare HFNC with CPAP. In particular, insufficient evidence is available for indications beyond bronchiolitis, such as asthma, pneumonia, postoperative support, and other pediatric conditions, necessitating additional randomized controlled trials.^25^

In the present survey, the following pattern regarding hypoxemia management was observed: when oxygenation targets were not achieved, most respondents reported increasing FiO_2_ as the initial interventional strategy. It should be noted that this approach is clinically plausible because it has been demonstrated as optimal.^26^ Physiologically, FiO_2_ primarily modifies oxygen delivery, whereas the flow rate also influences the washout of nasopharyngeal dead space, room air entrainment, and the degree of flow-related distending pressure.^27^ A recent review of flow settings in neonates and children showed that these effects are flow dependent and individualized rather than fixed, thereby partly explaining institutional differences in the preferred titration sequence.^28^ Thus, variability in initiation and adjustment practices may influence patient responses through differences in the physiologic support delivered, although this relationship was not directly assessed in the present study.

The monitoring practices reported by respondents generally focused on clinically relevant bedside parameters, i.e., pulse oximetry, respiratory rate, and work of breathing. This focus aligns with the current consensus that early HFNC reassessment should prioritize serial clinical examination rather than oxygen saturation alone.^5^^,^^19^ However, the observed variation in the reassessment frequency across emergency departments, PICUs, and wards may reflect differences in unit acuity, staffing models, escalation thresholds, and available monitoring infrastructure.^12^^,^^17^ Recent studies suggest that structured indices, such as the respiratory rate–oxygenation index or related respiratory scores, may help identify children at risk of HFNC failure as adjuncts to but not substitutes for repeated clinical assessment.^29^^,^^30^

Aerosol delivery during HFNC is another area where reported practice was common but not standardized. This inconsistency is important because current evidence supports the technical feasibility of this practice more than uniform clinical effectiveness.^5^^,^^19^ Bench and translational studies indicate that aerosol delivery through HFNC is strongly influenced by the gas flow, nebulizer type, and device position.^31^ Vibrating mesh nebulizers are generally superior to jet nebulizers because the increased gas flows used with jet nebulizers may reduce the dose delivered.^32^ Research into HFNC aerosol delivery also suggests that placing the nebulizer upstream of the humidifier can improve the delivery efficiency in many configurations, although the performance varies among devices and models.^33^^,^^34^ In the present survey, respondents reported variability in nebulizer placement, although most positioned the device on the dry side of the humidifier, which is a recommended method for minimizing drug degradation.^35^ By contrast, placement between the circuit and nasal cannula was reported less frequently. Collectively, these technical findings provide a plausible mechanistic explanation for the differences in the aerosol practices reported by our respondents.

An additional strength of the present study is that our findings suggest contextual determinants of HFNC variability that extend beyond bedside physiology. The initiation of HFNC, its escalation or weaning, and whether aerosol therapy is delivered in-line or after temporary HFNC discontinuation are probably determined by differences in institutional protocols, implementation culture, clinician training, staffing, and equipment availability. Indeed, this interpretation is supported by demonstrations in recent quality improvement and multicenter studies that protocolized HFNC initiation and weaning can reduce HFNC use, shorten the duration of therapy, and decrease the length of hospital stay without worsening key balancing measures. In particular, hospitals with restrictive HFNC policies further confirm this pattern as they have decreased initiation rates and treatment durations.^36^^,^^37^

Overall, our findings suggest that HFNC has become an established component of pediatric respiratory support in western KSA, but that substantial variability persists in how the therapy is defined, initiated, monitored, and combined with aerosol delivery. Rather than implying that this variability directly determines clinical effectiveness, our data indicate areas where standardization may be beneficial. Therefore, the development of pediatric HFNC guidance in KSA should focus on technical settings but also on operational elements, such as indications for initiation, reassessment intervals, escalation criteria, aerosol delivery procedures, and staff training requirements. A national framework adapted to local resource differences could help reduce unwarranted variation and promote more consistent HFNC care processes across institutions.

Future research should expand this work to a national level to determine whether similar patterns exist across KSA. Our survey provides valuable insights into current RT practices related to pediatric HFNC use but future research should move toward prospective evaluation of how specific HFNC protocols perform in Saudi pediatric settings. In particular, multicenter studies linking protocol characteristics with patient-level outcomes, resource utilization, and escalation patterns would help determine the most important components of HFNC care that need to be standardized.

The present study had several limitations that should be acknowledged. First, the survey relied on self-reported practices and did not involve direct observation of clinical care or assessment of patient outcomes. Similar to all survey-based research, the responses may have been influenced by social desirability or recall bias, although anonymization might have mitigated these effects. Second, the recruitment strategy may have resulted in the overrepresentation of RTs with greater interest in HFNC or higher confidence in their knowledge. Finally, the relatively small sample size, which was largely due to time constraints during data collection, limits the representativeness of the sample and restricts the generalizability of the findings. Accordingly, the results should be interpreted with caution.

## Conclusion

In this study, it was demonstrated that HFNC therapy is widely utilized in pediatric care, particularly in PICUs across the western region of KSA. However, the findings obtained highlight an urgent need to develop national clinical guidelines or protocols for pediatric HFNC therapy to promote best practices and optimize resource use across the healthcare system.

## Authors contributions

AMA - conceived and designed the study. AMA - conducted and supervised data collection. AMA - analyzed and interpreted data. AMA - wrote initial and final draft. The author critically reviewed and approved the final draft and is responsible for the content and similarity index of the manuscript. AMA - submitted the paper for publication. All authors have critically reviewed and approved the final draft and are responsible for the content and similarity index of the manuscript.

## Source of funding

This research did not receive any specific grant from funding agencies in the public, commercial, or not-for-profit sectors.

## Conflict of interest

The authors have no conflict of interest to declare.
